# Maternal Undernutrition in Focus: Determinants among pregnant and lactating women in Kurrum District, Pakistan: A Community-based Study

**DOI:** 10.12669/pjms.42.2.13164

**Published:** 2026-02

**Authors:** Ijaz Habib, Sheraz Fazid, Saima Aleem, Abdullah Abdulmohsen Alsabaani Alshehri, Zia Ul Haq

**Affiliations:** 1 Ijaz Habib PhD Scholar, Institute of Public Health & Social Sciences World Food Program, Pakistan. Institute of Public Health & Social Sciences, Khyber Medical University, Peshawar, Pakistan; 2 Sheraz Fazid Institute of Public Health & Social Sciences, Institute of Public Health & Social Sciences, Khyber Medical University, Peshawar, Pakistan; 3 Saima Aleem Institute of Public Health & Social Sciences, Institute of Public Health & Social Sciences, Khyber Medical University, Peshawar, Pakistan; 4 Abdullah Abdulmohsen Alsabaani Alshehri King Khalid University, Saudi Arabia; 5 Zia Ul Haq Institute of Health and Wellbeing, University of Glasgow, UK. Institute of Public Health & Social Sciences, Khyber Medical University, Peshawar, Pakistan

**Keywords:** Determinants, Khyber Pakhtunkhwa, Maternal undernutrition, Multisectoral, Merged districts, Pakistan, Pregnant and lactating women

## Abstract

**Background & Objective:**

Undernutrition during pregnancy and lactation affects infant growth, development, and resistance to infection. This study aimed to assess maternal undernutrition in the tribal district of Kurrum, Khyber Pakhtunkhwa, Pakistan, and its various contributing factors.

**Methodology:**

We conducted a community-based cross-sectional study nested within our registered trial (ISRCTN94319790) in Upper Kurrum (January 2018 to December 2020). Pregnant and lactating women were selected through two-stage stratified random sampling. Trained enumerators collected socioeconomic and demographic data using a pre-tested questionnaire. MUAC was measured with standardized tapes, and multivariate regression was performed in STATA 14.

**Results:**

The mean MUAC measurement of 2,195 PLWs was 26.37 cm (+- 3.49 SD). The overall prevalence of MUAC based undernutrition was 16.3% (95% CI: 14.8%–17.9%) and significantly associated with younger maternal age (p-value <0.001), low educational status (p-value <0.001), and open drainage toilet used (p-value 0.04) in the household. On multivariate regression analysis there was an increased likelihood of undernutrition in PLWs with lower maternal age i.e. <=20 (OR 0.38, 0.27-0.54 95% CI). Undernutrition was more likely among PLWs belonging to households with only one earning member (OR 1.63, 95% CI 1.19, 1.98) and using water treatment facility compared to none (OR 1.95, 95% CI 1.49, 2.55).

**Conclusion:**

Maternal undernutrition in Kurrum exceeds national averages and is strongly influenced by maternal age, socioeconomic vulnerability, sanitation, and education. Strengthening maternal nutrition programs, improving WASH conditions, and addressing economic barriers are essential for reducing undernutrition in high-burden districts.

## INTRODUCTION

Optimal nutrition during pregnancy and lactation is essential for a woman’s health and for the healthy development of her child. Nutrition during pregnancy and the first two years of a child’s life plays a decisive role in shaping growth and development, a phase frequently termed the first 1,000 days.^1^ When mothers are undernourished during this time, infants are more likely to experience restricted fetal growth, low birth weight, premature delivery, and also later developmental difficulties.^2^,^3^ Despite global attention, progress in improving maternal nutrition has been slow. Over the past two decades, reductions in maternal undernutrition have been uneven, and large differences persist between regions.^4^,^5^ Recent analyses, including the 2021 Lancet Maternal and Child Undernutrition series, continue to show that maternal undernutrition is a key contributor to poor pregnancy outcomes such as preterm birth.^6^ These reports also emphasize the importance of strengthening antenatal nutrition care, including balanced energy-protein supplements and improved micronutrient support.^7^,^8^ Pakistan continues to carry a considerable burden of maternal undernutrition. According to the National Nutrition Survey (NNS) 2018, 14.2% of women of reproductive age were underweight, with even poorer indicators reported from Khyber Pakhtunkhwa’s merged districts (MDs), where stunting reaches 48.3% and maternal undernutrition remains a major concern.^9^,^10^ At the same time, the province faces a dual challenge, as underweight and overweight coexist within the population. Maternal undernutrition also contributes to higher infant and maternal mortality across KP.^11^

These concerns are well known and has informed the development of Pakistan’s Maternal Nutrition Strategy (2022–2027), which highlights maternal nutrition as an essential pathway for improving outcomes during pregnancy and early childhood.^12^ However, district-level evidence from the high-burden and underserved regions, such as the merged tribal districts of KP, remains scarce. Tribal merged districts are characterized by chronic poverty, low female literacy, poor WASH infrastructure, and limited access to quality health services. These factors collectively aggravate nutritional vulnerability among pregnant and lactating women (PLWs).

## METHODOLOGY

**Fig. 1:**
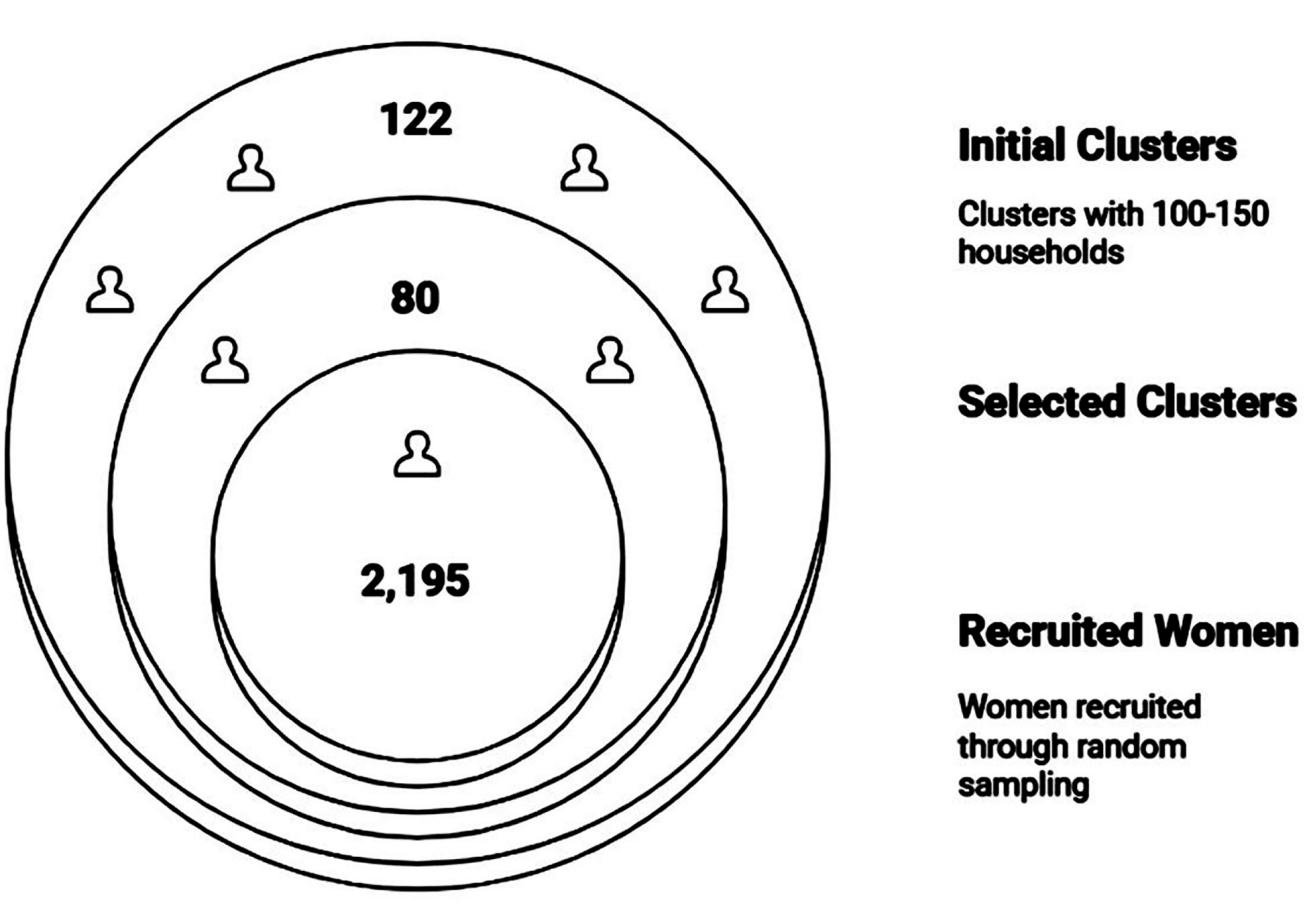
Recruitment of Study Participants.

Our Kurrum cross-sectional study is part of a larger community-based trial (The trial is registered (ISRCTN94319790), carried out in the district between January 2018 to December 2020. The study was undertaken to estimate the burden and determinants of maternal undernutrition in a low-resource setting. The study population included women in early pregnancy and mothers who were breastfeeding young infants up to six months postpartum, with this restriction applied to minimize heterogeneity in nutritional status. The study areas were selected using WHO EPI micro-plans, which are commonly used by local health staff for household mapping. In total, 122 clusters were identified, and from these, 80 were chosen through random selection. We then used the existing Lady Health Worker household lists to enroll the eligible women. A total of 2195 females (approx. 27 per cluster) were recruited through random sampling (Fig.1).

### Ethical approval:

It was obtained from the Khyber Medical University (Ref. No.: DIR/KMU-EB/SP/000427; dated: August 28, 2017Advance Study Review Board. Written informed consents were taken from the participants, and their privacy and confidentiality were strictly maintained throughout the study and after completion of data collection.

### Informed Consent statement:

Informed consent was obtained from all subjects involved in the study.

**Fig. 2:**
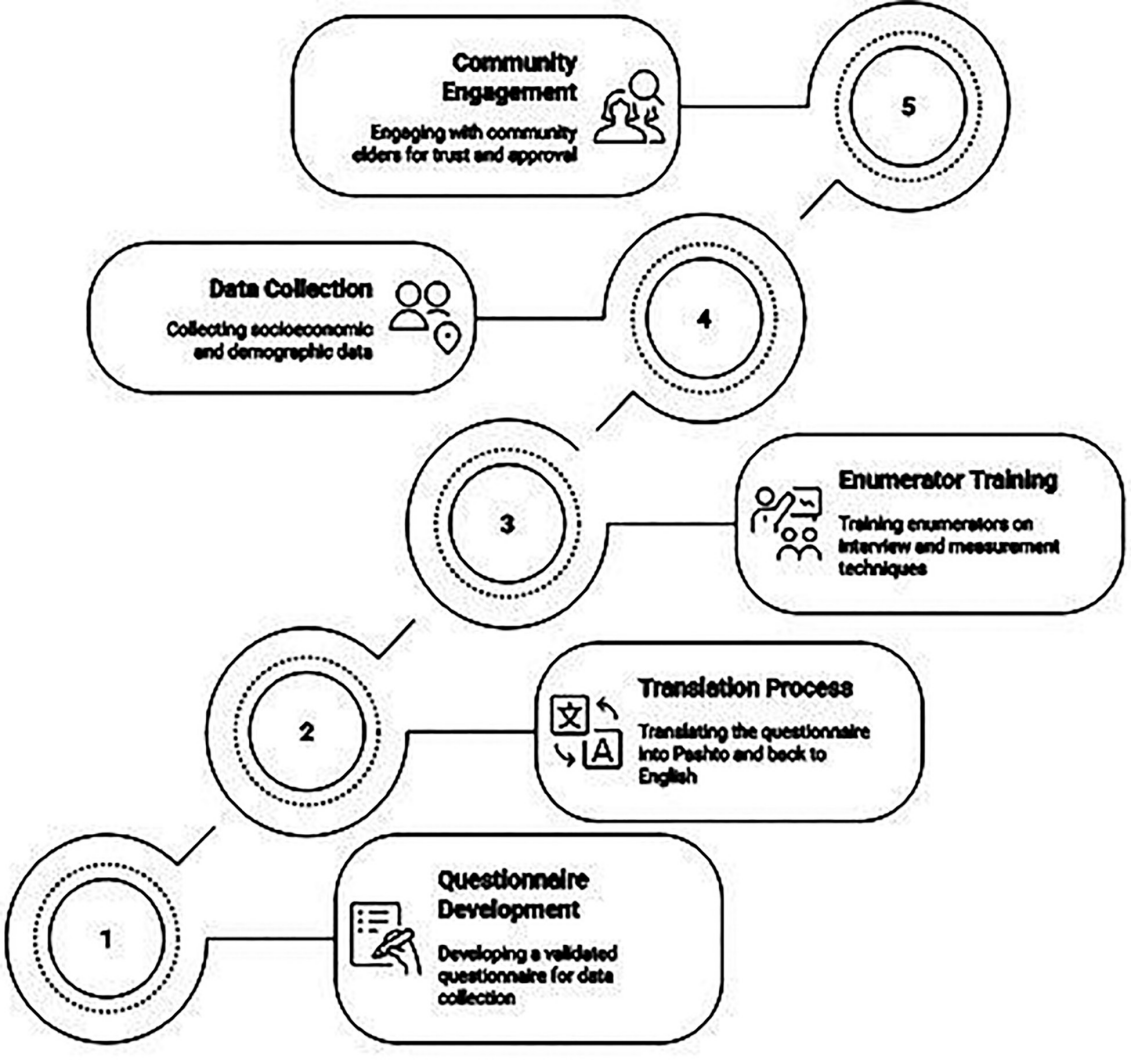
Overview of Research Methodology.

Following the detailed methodology regarding the tool development, its translation and training of the field team (Fig.2), the data were collected by female field teams, who worked with the local Lady Health Workers (LHWs) of Department of Health. The trained females visited households and carried out interviews in a private setting. The team used pre-tested questionnaires capturing socioeconomic, reproductive and demographic factors. The tool was developed in English, translated into Pashto and back translated to preserve conceptual fidelity. Enumerators completed standardized training in interviewing and anthropometry; calibrated MUAC tapes were used.

Socioeconomic status (SES) was assessed by the Pakistan poverty assessment scorecard with categories high (>66%), medium (34–66%) and low (<34%) probability thresholds. Additional variables included age, years of schooling, marital and employment status, husband’s education and occupation, family structure (nuclear/joint), toilet type (flush to open drain/septic/sewerage/other), and reported household water treatment.

### Nutritional status:

The midpoint of the left upper arm was identified, and the circumference was measured and recorded to 0.1 cm precision, between acromion and olecranon using inelastic tapes. Measurements near the threshold were repeated, with ~10% re-measured for QA. Undernutrition was defined as MUAC <23 cm, consistent with global guidance.^13^,^14^

### Statistical analysis:

Data were entered daily using electronic forms with in-built validation checks and analyzed in Stata 14. For descriptive analysis, continuous variables were summarized using mean values with corresponding standard deviations, while categorical variables were presented as counts and percentages. Group differences were examined using the chi square test. Univariate and multivariable logistic regression models were used to identify predictors of undernutrition (MUAC <23 cm), reporting odds ratios (ORs) and 95% confidence intervals (CIs). Cluster-robust standard errors accounted for the sampling design. A threshold of < 0.05 was used to indicate statistical significance.

## RESULTS

As presented in Table-I, a total of 2,195 pregnant and lactating women (PLWs) were enrolled in the study. The mean Mid-Upper Arm Circumference (MUAC) was 26.37 ± 3.49 cm. Overall, 16.3% (n = 356) met the criterion for undernutrition (MUAC < 23 cm), corresponding to a prevalence of 16.3% (95% CI: 14.7%–17.8%), while 83.7% (n = 1,839) were classified as normal.

**Table-I:** Socioeconomic characteristics of the participating Pregnant and Lactating women (PLW) by MUAC categories (n=2,195).

Variable	Undernourished N= 356 (16.3%) n (%)	Normal N= 1839 (83.7%) n (%)	p-Value
Living structure			
Single	63 (17.7%)	316 (17.2%)	0.8
Joint	293 (82.3%)	1523 (82.8%)	
Household members working in elementary occupation (non-skill workers / daily wages)			
None	68 (19.1%)	302 (16.4%)	0.002
One	149 (41.9%)	632 (34.4%)	
Two or More	139 (39.1%)	905 (49.2%)	
Highest education of female head			
Less than class 1	307 (86.24%)	1,611 (87.6%)	0.04
No education	13 (3.65%)	102 (5.55%)	
Class 1 or higher	36 (10.11%)	126 (6.85%)	
Poverty Level			
20 or below (Poorest)	28 (7.87%)	187 (10.17%)	0.03
Intermediate	279 (78.37%)	1,477 (80.32%)	
Richest	49 (13.76%)	175 (9.52%)	
Type of toilet			
Flush connected to public sewerage	13 (3.65%)	114 (6.2%)	
Flush connected to open drain/Septic tank	335 (94.1%)	1,653 (89.89%)	0.04
Other	8 (2.25%)	72 (3.92%)	
Age of the women (Years)			
<=20	58 (16.29%)	111 (6.04%)	<0.001
21-30	225 (63.2%)	1,132 (61.56%)	
31-40	71 (19.94%)	534 (29.04%)	
40 & above	2 (0.56%)	62 (3.37%)	
Educational status			
No formal education	208 (59.94%)	1,342 (73.78)	<0.001
Primary	43 (12.39%)	158 (8.69%)	
Middle	25 (7.2%)	86 (4.73%)	
Bachelors/above	53 (15.27%)	154 (8.47%)	
Graduate	18 (5.19%)	79 (4.34%)	
Water cleaning measures			
No	245 (68.82%)	1,506 (81.89%)	<0.001
Yes	111 (31.18%)	333 (18.11%)	
Household food insecurity			
No	333 (93.54%)	1,704 (92.66%)	0.5
Yes	23 (6.46%)	135 (7.34%)	

In adjusted analyses, only a few factors remained independently associated with undernutrition. PLWs from households with only one earning member and those reporting household water treatment were more likely to be undernourished, whereas older maternal age was protective. No other socioeconomic or WASH-related variables remained significant.

Comparisons between undernourished and normal PLWs showed several significant differences. Family structure was similar across both groups, with most women living in joint households. Household occupational composition differed (p = 0.002), with undernourished women more often coming from households with no members in elementary occupations, and normal women more frequently from households with two or more such workers. The educational level of the female household head also varied (p = 0.04), with a slightly higher proportion of undernourished women belonging to households where the female head had schooling beyond class 1.

Poverty categories differed modestly (p = 0.03). In sanitation, a flush toilet connected to an open drain or septic tank was more common among undernourished women (94.1% vs 89.9%, p = 0.04). Younger age showed a strong association; PLWs aged ≤20 years were more frequently undernourished (p < 0.001). Maternal education also differed significantly (p < 0.001), and reporting household water treatment was notably higher among undernourished women (31.2% vs 18.1%, p < 0.001). No meaningful differences were observed for household food insecurity.

As summarized in Table-II, univariate regression showed significant associations between undernutrition and household occupation type, female head’s education, poverty category, maternal age, reported water treatment, and food insecurity.

**Table-II:** Logistic regression analysis of the Pregnant and lactating mother’s characteristics associated with having undernutrition (MUAC<23) (n=2,195).

	Un Adjusted			Adjusted		
Variable	OR	p-value	95% CI	OR	p-value	95% CI
Single	Reference					
Joint	0.95	0.82	0.72-1.30	0.95	0.39	0.61-1.21
***Mother Age***						
<=20	Reference					
21-30	0.38	<0.001	0.27-0.54	0.35	<0.001	0.24-0.51
31-40	0.25	<0.001	0.17-0.38	0.24	<0.001	0.16-0.37
40& above	0.06	<0.001	0.01-0.26	0.06	<0.001	0.01-0.27
***Water Treatment***						
Yes	2.05	<0.001	1.59-2.64	1.95	<0.001	1.49-2.55
No	Reference					
***Household Food Insecurity***						
Yes	0.65	<0.001	0.51-0.82	0.72	0.01	0.56-0.93
No	Reference					
***Elementary Occupation*** Household head occupation type* (daily or contract wage labor that does not require training)						
Two or More		Reference				
One	1.53	0.00	1.19-1.98	1.63	<0.001	1.23-2.16
None	1.47	0.02	1.07-2.01	1.33	0.12	0.93-1.92
***Female Head Education***						
Less than class 1 or no	Reference					
No female head	0.67	0.18	0.37-1.21	0.71	0.27	0.38-1.31
Class 1 or higher	1.50	0.04	1.02-2.21	1.08	0.73	0.70-1.67
***Poverty***						
20 or below	0.79	0.28	0.52-1.20	1.16	0.52	0.74-1.81
Intermediate	Reference					
Richest	1.48	0.02	1.05-2.09	1.03	0.87	0.71-1.51
***Toilet Facility***						
Other						
Flush connected to open drain	1.82	0.11	0.87-3.82	1.54	0.27	0.72-3.31
Flush connected to public sewerage	1.03	0.96	0.41-2.60	1.01	0.98	0.38-2.67
***Female Education***						
Illiterate	0.68	0.16	0.40-1.16	0.85	0.56	0.48-1.49
Primary	1.19	0.57	0.65-2.20	1.27	0.46	0.67-2.41
Middle	1.28	0.48	0.65-2.51	1.13	0.73	0.56-2.31
Bachelors/above	1.51	0.18	0.83-2.75	1.47	0.22	0.79-2.75
Masters	Reference					

**Fig.3:**
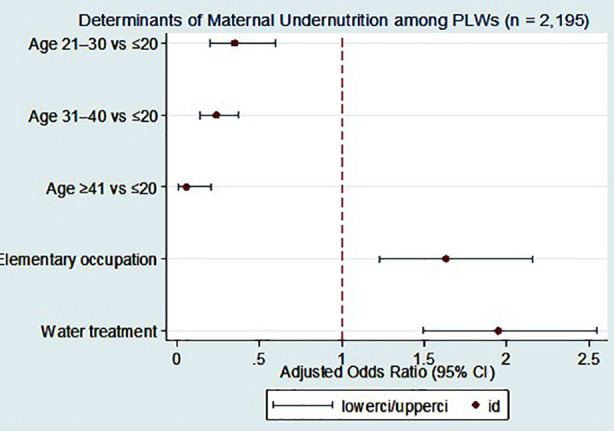
Determinants of maternal undernutrition among pregnant and lactating women in District Kurrum. Adjusted odds ratios (AORs) with 95% confidence intervals for predictors of undernutrition (MUAC < 23 cm) among PLWs (n = 2,195).

Multivariate results (Table-II; Fig.3) identified two independent predictors of undernutrition: having one household member in an elementary occupation (AOR 1.63; 95% CI 1.23–2.16) and reporting water treatment (AOR 1.95; 95% CI 1.49–2.55), both p < 0.001. Compared with PLWs aged ≤20 years, older age groups had lower odds of undernutrition; 21-30 years (AOR 0.35; 95% CI 0.20–0.60), 31–40 years (AOR 0.24; 95% CI 0.14–0.37), and ≥40 years (AOR 0.06; 95% CI 0.01–0.21), all p < 0.001. Fig.1 presents the adjusted odds ratios with 95% CIs for key determinants.

## DISCUSSION

This study provides evidence on maternal undernutrition in Kurrum, one of KP’s newly merged districts. The prevalence of 16.3% exceeds national NNS 2018 estimates for women of reproductive age, reaffirming that NMDs carry a disproportionate burden.^9^ Our findings also diverge from national patterns: unlike NNS 2018, where household poverty and food insecurity were dominant predictors, the key determinants in Kurrum were young maternal age, low education, limited occupational security, and WASH-related exposures. The Kurrum district characteristics highlight that district-level realities do not always represent the national trends and that localized programming is essential based on actual contextual needs. The strong age gradient, with markedly higher undernutrition among the very young mothers in Kurrum, is consistent with the evidence that adolescents face greater nutritional vulnerability due to concurrent growth demands, limited autonomy, and reduced access to timely care.^15^ It tell us that addressing nutritional needs earlier, including before and between pregnancies, is important for settings like Kurrum, where early marriage and adolescent pregnancy remain common.^16^ Comparative evidence from low-income settings also shows Kurrum like vulnerability patterns in young pregnant women.^17^,^18^

Education also emerged as an important correlate. Lower schooling among women and female household heads was associated with higher undernutrition, in line with research linking education to improved health practices.^18^,^19^ For KP, this supports a dual approach: targeted counselling and ANC/PNC service integration for current PLWs, alongside long-term investments in girls’ education to strengthen structural determinants of nutrition.

WASH conditions contributed meaningfully to the risk profile and poor sanitation was seen more common among undernourished women. The association with reported household water treatment likely reflects a reverse causality factor. Water treatment is more often practiced in Kurrum, in households which are already exposed to unsafe water. Studies from similar settings, including Pakistan highlight how environmental and sanitary contamination relates to the nutrition vulnerabilities in resource-limited settings.^20^ Our findings are consistent with the local and global evidence, reiterating the need to integrate water safety, sanitation improvements, and hygiene promotion into maternal nutrition programming.^4^,^14^

Economic constraints showed up clearly in our study findings. Women from households with only a single earner, often in low-paid, unstable work, were more likely to be undernourished. This pattern is in line with both national and international guidance, which stresses that nutrition interventions work best when paired with support for household income and livelihoods.^2^,^3^,^5^,^7^,^21^

In Pakistan, the BISP Nashonuma initiative already offers a platform for reaching nutritionally vulnerable mothers. The program has shown that targeted support can be delivered effectively, and it could have even greater impact if combined with WASH improvements, adolescent nutrition efforts, and focused counselling in districts with high levels of undernutrition like Kurrum.^12^,^22^-^24^

### Policy implications:

Kurrum study findings point towards the need for district-specific approaches.

**Fig.4:**
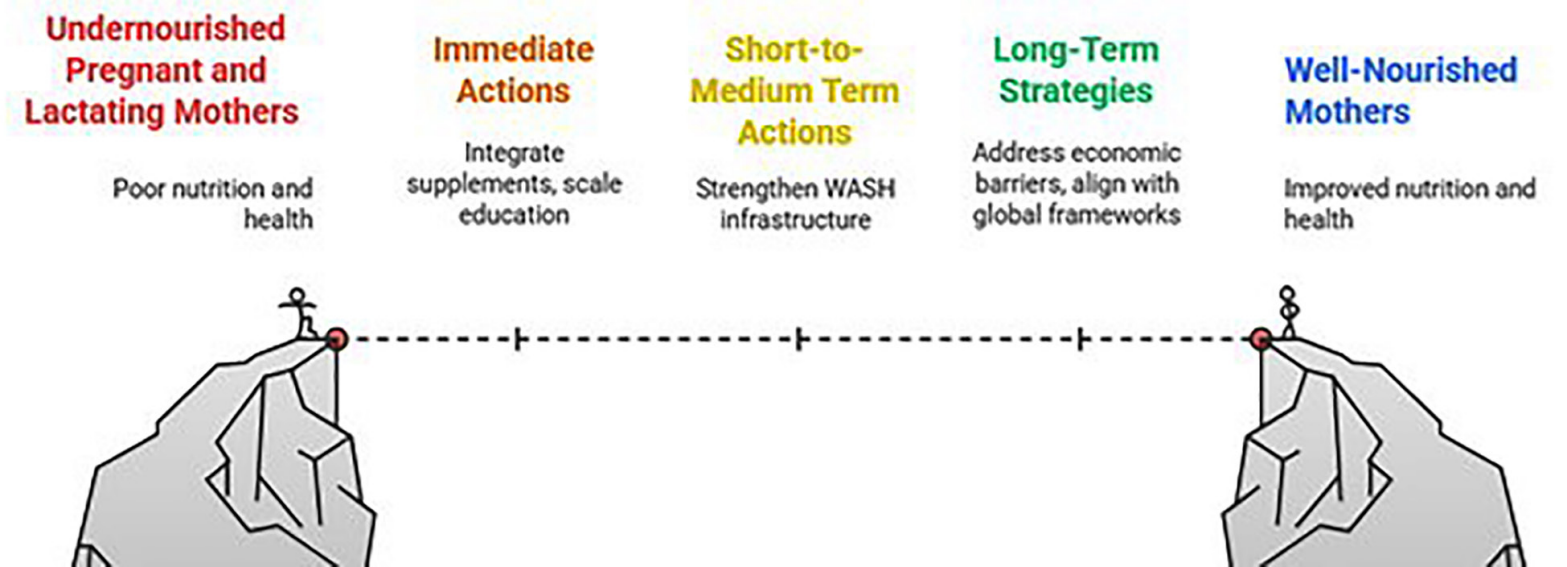
Policy Implications for Improving Pregnant and Lactating Female Nutrition.

Strengthening support for younger mothers, improving ANC and PNC nutrition counselling, and addressing WASH gaps should continue alongside social protection systems such as BISP Nashonuma, which can help cushion income instability at the household level (Fig.4).

### Strengths and limitations:

Our Study was based on a large community sample, standardized MUAC measurement, two-stage cluster sampling, and analytic methods consistent with the study design. As a cross-sectional study, causal inference is limited, and some determinants (e.g., dietary diversity, morbidity) were not measured. Also the single district setting may constrain generalizability, though the findings remain applicable to similar merged tribal districts contexts.

## CONCLUSION

Kurrum study highlights the complex determinants of undernutrition among pregnant and lactating women, exceeding the national averages. Our findings point to an urgent need for better maternal education and nutrition awareness, improved sanitation, and stronger socioeconomic support systems. Strategic investments in these areas can substantially reduce the burden of maternal undernutrition and serve as a model for similar underserved districts in Pakistan.

### Future research:

Kurrum findings suggest longitudinal studies, to clarify further pathways linking maternal malnutrition with maternal age, education, WASH and socioeconomic vulnerabilities.

In Kurrum, maternal undernutrition reflects overlapping biological and social disadvantages. Younger and less resourced poor mothers remain most at risk. There is a need for improved counselling to prevent early age marriages, promote better sanitation, and invest in targeted socioeconomic activities.
